# HLA genotyping and clinical characteristics of early-onset and late-onset anti-LGI1 encephalitis: a single-center cohort study in China

**DOI:** 10.3389/fimmu.2026.1758914

**Published:** 2026-04-01

**Authors:** Pinfei Ni, Siyuan Fan, Han Zhang, Lin Bai, Zhuo Yang, Xinzhuang Yang, Haitao Ren, Qiang Lu, Meng Xiao, Hongzhi Guan

**Affiliations:** 1 Department of Neurology, Peking Union Medical College Hospital, Chinese Academy of Medical Sciences & Peking Union Medical College, Beijing, China; 2 Department of Laboratory Medicine, Peking Union Medical College Hospital, Chinese Academy of Medical Sciences & Peking Union Medical College, Beijing, China; 3 Center for bioinformatics, National Infrastructures for Translational Medicine, Institute of Clinical Medicine, Peking Union Medical College Hospital, Chinese Academy of Medical Sciences & Peking Union Medical College, Beijing, China

**Keywords:** A*02:01, autoimmune encephalitis, DRB1*07:01, human leucocyte antigen, leucine-rich glioma-inactivated 1

## Abstract

**Objectives:**

To investigate the human leukocyte antigen (HLA) associations and clinical characteristics of anti-Leucine-rich glioma-inactivated 1 (LGI1) encephalitis in a Chinese cohort, focusing on potential differences between early-onset and late-onset patients.

**Methods:**

Eighty patients diagnosed with anti-LGI1 encephalitis at Peking Union Medical College Hospital between the years 2016 and 2024 were included. Patients were stratified into early-onset (<50 years, n=22) and late-onset (≥50 years, n=58) groups for clinical features analysis. High-resolution NGS HLA genotyping was performed and compared with 984 healthy controls. Logistic and linear regressions were used to analyze disease susceptibility, age of onset associations, and prognostic factors.

**Results:**

Compared with the late-onset group, the early-onset group more frequently presented with generalized tonic-clonic seizures (GTCS) as the initial symptom (45.5% *vs*. 10.3%, *post-hoc p* = 0.001), and a lower frequency of psychiatric symptoms (*p* = 0.047) and amnesia (*p* = 0.002) during the disease course, presenting with lower mRS scores at onset (*p* = 0.020). Genetically, DRB1*07:01 was confirmed as the primary risk allele (OR = 10.4, 95% CI 6.01-18.02, *p^c^* = 6.78×10^−15^). DRB1*09:01(OR = 3.67, 95% CI 2.17-6.20, *p^c^* = 1.16×10^-5^) and DQB1*03:03 (OR = 3.25, 95% CI 1.98-5.33, *p^c^* = 1.32×10^-5^) were identified as novel secondary risk alleles in this Chinese cohort. Importantly, specific HLA genotypes were significantly associated with the age of onset. A*02:01 was linked to an earlier onset (β = -9.26; *p^c^* = 0.019) and served as a risk factor for the early-onset group, while DRB1*07:01 was associated with a later onset (β = 13.44, *p^c^* = 0.006). Diagnostic delay (OR = 1.01, 95% CI 1.00-1.02, *p* = 0.037) and GTCS (OR = 5.01, 95% CI 1.11-22.66, *p* = 0.036) were independent predictors of poor prognosis.

**Conclusions:**

This study suggests that early-onset and late-onset anti-LGI1 encephalitis may possess differential immunogenetic and clinical profiles. In addition to the broad susceptibility conferred by DRB1*07:01, the presence of A*02:01 is significantly associated with an earlier onset age. These findings provide insights into the role of HLA genotyping in accounting for the observed differences between early-onset and late-onset patients.

## Introduction

Since the identification of Leucine-rich glioma-inactivated 1 (LGI1) autoantibodies in 2010 in patients with voltage-gated potassium channel-complex antibodies positive limbic encephalitis (LE), anti-LGI1 encephalitis has emerged as one of the most common forms of autoimmune LE (1, 2). LGI1 is a secreted neuronal protein that interacts with presynaptic and postsynaptic membrane proteins (ADAM23/22), to form a trans-synaptic complex which modulates presynaptic voltage-gated potassium channels Kv1.1 and postsynaptic α-amino-3-hydroxy-5-methyl-4-isoxazolepropionic acid (AMPA) receptor function (3). Anti-LGI1 encephalitis typically affects middle-aged and older adults and clinically presents with amnesia, psychiatric symptoms, and seizures. Faciobrachial dystonic seizures (FBDS) are considered a pathognomonic manifestation (4–6). Most patients show favorable outcomes after immunotherapy (7–9).

Recent studies on the hypothesized autoimmune mechanisms underlying anti-LGI1 encephalitis have increasingly focused on genetic predispositions to autoimmunity. The human leukocyte antigen (HLA) gene group is considered the most significant factor in this context. HLA genes, which constitute the human major histocompatibility complex (MHC), encode antigen-presenting proteins on the surface of the cells and play a central role in immune responses (10). A strong association between anti-LGI1 encephalitis and HLA genes has been consistently demonstrated (11–15). In Korean and Caucasian populations, the haplotype DRB1*07:01-DQB1*02:02 is the most well-defined genetic risk marker to date, with a reported carriage rate of up to 88-91% in patients compared to 12-26% in healthy individuals (14). Additionally, DRB1*04:02 has been identified as a secondary risk allele, particularly enriched among DRB1*07:01-negative patients (15). DRB1*07:01-positive patients are more prone to exhibiting characteristic FBDS, whereas DRB1*07:01-negative patients show a lower age of onset (45 *vs*. 65 years) and a higher proportion of females (75% *vs*. 40%) (15).

In Chinese patients with anti-LGI1 encephalitis, Zhen Hong et al. reported DRB1*03:01-DQB1*02:01 as a novel susceptibility haplotype, but failed to observe the widely reported international association with DRB1*07:01 (16). Furthermore, patients carrying the DRB1*03:01 allele were predominantly female and presented with earlier disease onset and better immunotherapy responses (17). To investigate whether early-onset patients exhibit distinct clinical and genetic characteristics, this study will investigate the HLA susceptibility genes in Chinese anti-LGI1 encephalitis patients and compare the clinical and genetic features between early-onset and late-onset patients.

## Materials and methods

### Patients and clinical data

Eighty Chinese patients diagnosed with anti-LGI1 encephalitis were recruited consecutively from 2016 to 2024 at Peking Union Medical College Hospital, a tertiary referral center for autoimmune neurological disorders (18). Inclusion criteria were as follows (1): presence of anti-LGI1 antibody in serum or cerebrospinal fluid (CSF) and (2) at least 12 months of clinical follow-up. Detection of anti-LGI1antibodies in serum and CSF was performed using a commercial autoimmune encephalitis antibody panel containing NMDAR/LGI1/GABAR/CASPR2/GAD65/IgLON5/DPPX antibodies (EUROIMMUN, Lübeck, Germany) (18). Demographic and clinical data were retrospectively collected from hospital charts, including age of onset, first manifestation, clinical features that developed during the course of the disease, disability at onset using the modified Rankin Scale (mRS), diagnostic delay, hyponatremia, CSF positivity for LGI1-Abs, brain MRI (normal or medial temporal/basal ganglia hypersignal), acute immunotherapy (corticosteroids, IV immunoglobulin (IVIG)), chronic immunotherapy (mycophenolate or rituximab), mRS at 12-month follow-up. The study was approved by the institutional review board (K7384-K25C0191), and written informed consent was obtained from all patients.

### HLA genotyping data

1–2 mL of whole blood was collected from anti-LGI1 encephalitis patients at the follow-up period and sent to the Capital bio Technology Co., Ltd. (Beijing, China) for HLA genotyping. In brief, HLA genotyping was performed by the next-generation sequencing (NGS) method using the NGS-HLA gene high-resolution typing kit for 5 HLA loci (A, B, C, DRB1 and DQB1) on Illumina NovaSeq X-Plus platform. HLA typing results were obtained with the analysis of sequencing data (FASTQ files) using NGSengine software (GenDx, The Netherlands) and reported at a 4-digit level resolution.

The control group comprised 984 healthy subjects of Northern Han Chinese ethnic origin from the Shunyi Study in China (19). The data processing for the control group has been detailed in our previous publication (**Supplementary Table 1**) (20). In brief, raw whole-exome sequencing data were first processed for rigorous quality control. Adapter sequences and low-quality bases (Phred score Q < 20) were trimmed using fastp (v0.23.3), and reads that fell below a defined minimum length (100 bp) after trimming were discarded to ensure subsequent alignment reliability. HLA genotypes (A, B, C, DRB1, and DQB1) for controls were extracted from whole-exome sequencing (WES) data and reported at a 4-digit level resolution. Any ambiguous allele calls were excluded from the final reporting.

### Statistical analysis

Continuous variables are expressed as the mean (± standard deviation [SD]) in the case of a normal distribution or as median (interquartile range) and Categorical variables are expressed as the number (percentage). Clinical characteristics were compared between the early-onset (defined as age of onset < 50 years) and late-onset (age of onset ≥50 years) patients using the Mann-Whitney *U* test or unpaired Student t-test for continuous variables and Fisher’s exact test or χ2 test for Categorical variables.

Genetic data for five HLA loci (A, B, C, DRB1, and DQB1) were integrated from 80 patients and 984 healthy controls, all resolved to four-digit resolution. All HLA alleles were in Hardy-Weinberg equilibrium in the control group (**Supplementary Table 2**). For each multiallelic HLA locus, alleles were collapsed into a series of biallelic variants for association analysis.

To assess the degree of linkage disequilibrium (LD) between HLA loci, pairwise r² values were calculated (14). DRB1-DQB1 haplotypes were inferred using the Bridging Immuno Genomic Data Analysis Workflow Gaps (BIGDAWG) software package (v1.8), which implements functions from the R package haplo.stats. We performed dominant logistic regression models adjusted for sex and age to evaluate the association between HLA alleles and haplotypes and anti-LGI1 encephalitis. Only alleles and haplotypes observed in both the patient and control groups were included. Conditional analyses were performed by including the primary risk allele or haplotype as a covariate in the regression models to evaluate residual association signals within the HLA region. To account for multiple comparisons, we applied the false discovery rate (FDR) correction to the *p* values. Corrected p-values (*p^c^*) < 0.05 were considered statistically significant.

To explore the association between HLA genes and onset age of anti-LGI1 encephalitis, carrier frequencies of HLA alleles/haplotypes were compared between early-onset and late-onset patient groups using Fisher’s exact test or the χ² test, as appropriate. The median age at onset among carriers of each HLA allele/haplotype was also calculated and compared using the Mann-Whitney U test. Furthermore, a case-only linear regression analysis was performed with onset age as the outcome and HLA gene dosage (encoded per allele/haplotype) as predictors. The *p* values were corrected for multiple comparisons using the False Discovery Rate (FDR) method (denoted as *p^c^*).

Based on the mRS score at the 12-month follow-up, eighty patients were classified into two groups: good prognosis (mRS ≤ 2, n = 57) and poor prognosis (mRS > 2, n = 23). To search for factors associated with good or poor prognosis, a two-step approach was adopted. First, univariate binary logistic regression was performed for all clinical characteristics. Second, multivariate logistic regression analysis was applied. The dependent variable was good/poor prognosis as defined above. Independent variables incorporated into the multivariate model were those identified as statistically significant in the univariate analysis. Statistical analyses were computed using Python statistical software (V3.12.2) and the R statistical program (V4.5.0). The *p* or *p^c^* values < 0.05 were considered statistically significant.

## Results

### Clinical characteristics of patients according to age of onset

The clinical characteristics of 80 patients with anti-LGI1 encephalitis are summarized in **Table 1**. Patients were stratified into the early-onset group (<50 years) and late-onset group (≥50 years) based on a cutoff age of 50 years old at disease onset. The distribution of initial manifestations differed significantly between the two groups (*p* = 0.007). *Post-hoc* pairwise comparisons revealed a significantly higher prevalence of generalized tonic-clonic seizures (GTCS) in the early-onset group (45.5% *vs*. 10.3%, *post-hoc p* = 0.001). In contrast, differences regarding amnesia (9.1% *vs*. 24.1%, *post-hoc p* = 0.211) and psychiatric symptoms (0% *vs*. 3.4%, *post-hoc p* = 1.000) did not reach statistical significance.

**Table 1:** Clinical characteristics of patients with anti-LGI1 encephalitis in different onset age groups.

Characteristic	Overall cohort (n = 80)	early-onset group (n = 22)	late-onset group (n = 58)	*P* value
Men, n (%)	46 (57.5)	10 (45.5)	36 (62.1)	0.276
Median age, y	54.0 (48.3 - 64.0)	32.0 (23.8 - 42.5)	59.0 (53.0 - 66.8)	< 0.001
Median diagnostic delay, d	61.0 (31.0 - 99.8)	59.0 (32.0 - 68.5)	61.0 (31.0 - 140.5)	0.217
First symptom, n (%)				0.007
Psychiatric symptoms	2 (2.5)	0 (0)	2 (3.4)	
Amnesia	16 (20.0)	2 (9.1)	14 (24.1)	
FBDS	16 (20.0)	2 (9.1)	14 (24.1)	
GTCS	16 (20.0)	10 (45.5)	6 (10.3)	
Other seizures (CPS+SPS)	30 (37.5)	8 (36.4)	22 (37.9)	
Symptoms manifested during disease course, n (%)				
Psychiatric symptoms	22 (27.5)	2 (9.1)	20 (34.5)	0.047
Amnesia	66 (82.5)	13 (59.1)	53 (91.4)	0.002
FBDS	37 (46.3)	9 (40.9)	28 (48.3)	0.735
GTCS	40 (50.0)	14 (63.6)	26 (44.8)	0.211
Other seizures (CPS+SPS)	56 (70.0)	15 (68.2)	41 (70.7)	1
Sleep disorders	22 (27.5)	3 (13.6)	19 (32.8)	0.153
Autonomic symptoms	18 (22.5)	3 (13.6)	15 (25.9)	0.370
Personality changes	14 (17.5)	5 (22.7)	9 (15.5)	0.514
Hyponatremia, n (%)	24 (30.0)	6 (27.3)	17 (29.3)	1
mRS score at onset	3 (3 - 3)	3 (3 - 3)	3 (3 - 4)	0.020
CSF anti-LGI1 antibody positive (N = 58)^&^, n (%)	46 (79.3)	11 (61.1)	35 (87.5)	0.035
Abnormal MRI (N = 65); n (%)	55 (83.1)	12 (70.6)	42 (87.5)	0.138
Acute immunotherapy, n (%)				0.571
IVIG alone	9 (11.3)	2 (9.1)	7 (12.1)	
CS alone	19 (23.8)	7 (31.8)	12 (20.7)	
IVIG+CS	52 (65.0)	13 (59.1)	39 (67.2)	
Poor prognosis (mRS > 2)	23 (28.8)	4 (18.2)	19 (32.8)	0.313

FBDS, faciobrachial dystonic seizures; GTCS, generalized tonic-clonic seizures; CPS, complex partial seizures; SPS, simple partial seizures; mRS, modified rankin scale; CSF, cerebrospinal fluid; MRI, magnetic resonance imaging; IVIG, intravenous immunoglobulin; CS, corticosteroids.

Data are expressed as number (percentage) for categorical variables and as mean (standard deviation [SD]) for continuous variables in case of normal distributions and median (interquartile range [IQR]) otherwise. P values were compared between the early-onset (<50 years) and late-onset (≥50 years) groups. Unpaired Student’s t-test (when satisfying a normal distribution) or Mann-Whitney U test (when not satisfying a normal distribution) were used for continuous variables, and the χ2 test or Fisher's exact test were used for categorical variables.

^&^CSF anti-LGI1 antibody status was available for 58 patients; the remaining 22 patients without CSF data were confirmed positive in serum, satisfying the inclusion criteria.

During the disease course, the late-onset group also exhibited a higher frequency of psychiatric symptoms (34.5% *vs*. 9.1%; *p* = 0.047) and amnesia (91.4% *vs*. 59.1%; *p* = 0.002). Additionally, patients in the late-onset group had higher mRS scores at onset (3 [IQR 3-4], *vs*. 3 [IQR 3-3], *p* = 0.020) and a higher rate of CSF anti-LGI1 antibody positivity (35/40, 87.5%, *vs*. 11/18, 61.1%; *p* = 0.035). All patients received first-line immunotherapy after diagnosis, including corticosteroids alone (19/80, 23.8%), intravenous immunoglobulin (IVIG) alone (9/80, 11.3%), or combined corticosteroids and IVIG (52/80, 65.0%). At the 12-month follow-up, 23 patients were classified as having a poor prognosis based on mRS scores, including 4/22 (18.2%) in the early-onset group and 19/58 (32.8%) in the late-onset group (*p* = 0.313). No significant differences were observed between the two groups with respect to sex, time from symptom onset to diagnosis, hyponatremia, abnormal brain MRI findings, acute immunotherapy, or the proportion of patients with poor prognosis.

### HLA association in anti-LGI1 encephalitis

We analyzed the associations between HLA genes and disease susceptibility using a dominant logistic regression model adjusted for age and sex (**Table 2**). DRB1*07:01 exhibited the strongest association with anti-LGI1 encephalitis, being present in 77.5% of patients compared with 24.0% of healthy controls (OR = 10.4, 95% CI 6.01-18.02, *p*^c^ = 6.78×10^−15^), supporting its role as the primary genetic risk allele in this cohort. Several additional alleles were also associated with increased disease risk, including DQB1*02:02, C*06:02, B*13:02, DQB1*03:03, A*30:01, B*57:01, and DRB1*09:01 (**Table 2**). Conversely, DRB1*15:01 and DQB1*03:01 were less frequent among patients, suggesting potential protective effects.

**Table 2:** Logistic regression analysis of HLA genes in patients with different onset ages and healthy control participants.

HLA genes	Controls (n =984)	Cases (n = 80)			Model 1		Model 2	
		Overall cohort (n = 80)	Early-onset group (n = 22)	Late-onset group (n = 58)	OR (95% CI)	Corrected *p* value	OR (95% CI)	Corrected *p* value
DRB1*07:01	236 (24.0%)	62 (77.5%)	11 (50.0%)	51 (87.9%)	10.4 (6.01 - 18.02)	6.78E-15		
DQB1*02:02	211 (21.4%)	47 (58.8%)	6 (27.3%)	41 (70.7%)	5.03 (3.12 - 8.11)	1.81E-09	1.04 (0.47 - 2.27)	0.962
C*06:02	227 (23.1%)	45 (56.2%)	8 (36.4%)	37 (63.8%)	4.13 (2.57 - 6.63)	1.63E-07	1.43 (0.79 - 2.60)	0.358
B*13:02	156 (15.9%)	33 (41.2%)	3 (13.6%)	30 (51.7%)	3.65 (2.24 - 5.96)	6.11E-06	1.21 (0.68 - 2.18)	0.659
DQB1*03:03	260 (26.4%)	42 (52.5%)	10 (45.5%)	32 (55.2%)	3.01 (1.88 - 4.79)	8.12E-05	3.25 (1.98 - 5.33)	1.32E-05
A*30:01	152 (15.4%)	27 (33.8%)	2 (9.1%)	25 (43.1%)	2.72 (1.64 - 4.52)	1.88E-03	0.99 (0.55 - 1.77)	0.962
B*57:01	28 (2.8%)	10 (12.5%)	5 (22.7%)	5 (8.6%)	4.63 (2.12 - 10.14)	1.88E-03	2.04 (0.87 - 4.76)	0.178
DRB1*09:01	230 (23.4%)	34 (42.5%)	8 (36.4%)	26 (44.8%)	2.34 (1.46 - 3.76)	5.62E-03	3.67 (2.17 - 6.20)	1.16E-05
DRB1*15:01	300 (30.5%)	9 (11.2%)	3 (13.6%)	6 (10.3%)	0.29 (0.14 - 0.59)	7.09E-03	0.44 (0.21 - 0.91)	0.081
DQB1*03:01	357 (36.3%)	15 (18.8%)	5 (22.7%)	10 (17.2%)	0.42 (0.24 - 0.75)	3.75E-02	0.59 (0.32 - 1.07)	0.178

Model 1: Adjusted for age and sex.

Model 2: Further adjusted for the primary significant allele (DRB1*07:01) in addition to all variables in Model 1.

We then conducted conditional association analyses adjusting for the primary significant allele, DRB1*07:01(**Table 2**). The associations for DQB1*02:02, C*06:02, B*13:02, and A*30:01 were attenuated and became non-significant after conditioning, suggesting that their initial signals were possibly driven by linkage disequilibrium (LD) with DRB1*07:01 (**Supplementary Figure 1**). In contrast, DRB1*09:01(OR = 3.67, 95% CI 2.17-6.20, *p*^c^ = 1.16×10^−5^) and DQB1*03:03 (OR = 3.25, 95% CI 1.98-5.33, *p*^c^ = 1.32×10^−5^) remained significant, indicating that they represent secondary susceptibility loci. To further evaluate the relationship between these two alleles, we constructed a joint model including DRB1*09:01, DQB1*03:03, and DRB1*07:01. In this model, the independent effects of DRB1*09:01 (OR = 2.08, *p* = 0.108) and DQB1*03:03 (OR = 1.91, *p* = 0.134) were attenuated, likely due to the linkage disequilibrium between them (r^2^ = 0.83 in controls, 0.60 in patients).

Given the strong linkage disequilibrium between DRB1 and DQB1 loci, we further evaluated DRB1-DQB1 haplotypes (**Supplementary Table 3**). The DRB1*09:01-DQB1*03:03 (OR = 3.31, 95% CI 1.99-5.51, *p^c^* = 5.90×10^−6^) haplotype was enriched in patients and remained associated with disease susceptibility after adjustment for the primary risk haplotype DRB1*07:01-DQB1*02:02.

Although B*57:01, DRB1*15:01, and DQB1*03:01 were not in strong LD with DRB1*07:01, their associations were no longer significant after conditioning, possibly due to the small number of affected individuals in our cohort.

### Analysis of the association between HLA genes and age of onset

We have observed some early-onset patients exhibiting distinct clinical features compared to late-onset patients (**Table 1**). Hence, we compared the distribution of HLA genes between the early-onset and late-onset groups. The allele A*02:01 was significantly more frequent in the early-onset group (59.1% *vs*. 19.0%, *p*^c^ = 0.023) (**Table 3**). Patients carrying the allele A*02:01 exhibited a significantly earlier median age of onset compared to patients without this allele (49.0 [IQR 32.0-56.5] *vs*. 56.0 [IQR 51.0-66.0], *p*^c^ = 0.045**) (****Table 3**). Whereas DRB1*07:01(87.9% *vs*. 50.0%, *p*^c^ = 0.023), DQB1*02:02 (70.7% *vs*. 27.3%, *p*^c^ = 0.023), A*30:01 (43.1% *vs*. 9.1%, *p*^c^ = 0.025), B*13:02 (51.7% *vs*. 13.6%, *p*^c^ = 0.025), and C*06:02 (63.8% *vs*. 36.4%, *p*^c^ = 0.263) were preferentially enriched in the late-onset group (**Table 3**).

**Table 3:** Linear regression analysis of the association between HLA genes and age of onset.

Genes	Carriers of patients (n, %)			Median age (y, IQR)			Linear regression between HLA and onset age		
	Early-onset group (n = 22)	Late-onset group (n = 58)	Corrected *p* value	Patients with the gene	Patients without the gene	Corrected *p* value	β	95% CI	Corrected *p* value
A*02:01	13 (59.1%)	11 (19.0%)	0.023	49.0 (32.0 - 56.5)	56.0 (51.0 - 66.0)	0.045	-9.26	-14.99 - -3.52	0.019
DRB1*07:01	11 (50.0%)	51 (87.9%)	0.023	56.5 (51.0 - 66.0)	39.0 (27.5 - 53.2)	0.002	13.44	6.07 - 20.80	0.006
DQB1*02:02	6 (27.3%)	41 (70.7%)	0.023	59.0 (52.0 - 67.5)	50.0 (31.0 - 56.0)	0.001	13.84	7.47 - 20.22	0.001
A*30:01	2 (9.1%)	25 (43.1%)	0.025	64.0 (53.0 - 71.0)	52.0 (39.0 - 59.0)	0.002	12.75	6.41 - 19.08	0.003
B*13:02	3 (13.6%)	30 (51.7%)	0.025	62.0 (53.0 - 71.0)	51.0 (33.0 - 57.5)	0.001	13.9	7.94 - 19.85	0.001
C*06:02	8 (36.4%)	37 (63.8%)	0.263	56.0 (51.0 - 67.0)	51.0 (33.0 - 59.0)	0.035	9.85	3.78 - 15.92	0.019
DRB1*07:01-DQB1*02:02	6 (27.3%)	41 (70.7%)	0.023	59.0 (52.0 - 67.5)	50.0 (31.0 - 56.0)	0.001	13.84	7.47 - 20.22	0.001
A*30:01-B*13:02-C*06:02-DRB1*07:01-DQB1*02:02	2 (9.1%)	25 (43.1%)	0.025	64.0 (53.0 - 71.0)	52.0 (39.0 - 59.0)	0.002	13	6.22 - 19.79	0.004

Linear regressions of onset age on HLA alleles show that A*02:01 was the only allele that negatively associated with onset age in anti-LGI1 encephalitis (β = -9.26; *p*^c^ = 0.019). While DRB1*07:01(β = 13.44; *p*^c^ = 0.006), DQB1*02:02(β = 13.84; *p*^c^ = 0.001), A*30:01(β = 12.75; *p*^c^ = 0.003), B*13:02 (β = 13.90; *p*^c^ = 0.001) and C*06:02 (β = 9.85; *p*^c^ = 0.019) were strongly associated with a later age of onset (**Table 3**). Furthermore, we found that all patients carrying the allele A*30:01 concurrently possessed the B*13:02, C*06:02, DRB1*07:01 and DQB1*02:02 alleles, due to the linkage disequilibrium (**Supplementary Figure 1**). Hence, we constructed the haplotype A*30:01-C*06:02- B*13:02-DRB1*07:01-DQB1*02:02 and found it was also associated with a later age of onset (β = 13; *p*^c^ = 0.004) (**Table 3**). Beta (β) represents the average change in the age of onset (in years) associated with per copy of HLA alleles.

**Figure 1:**
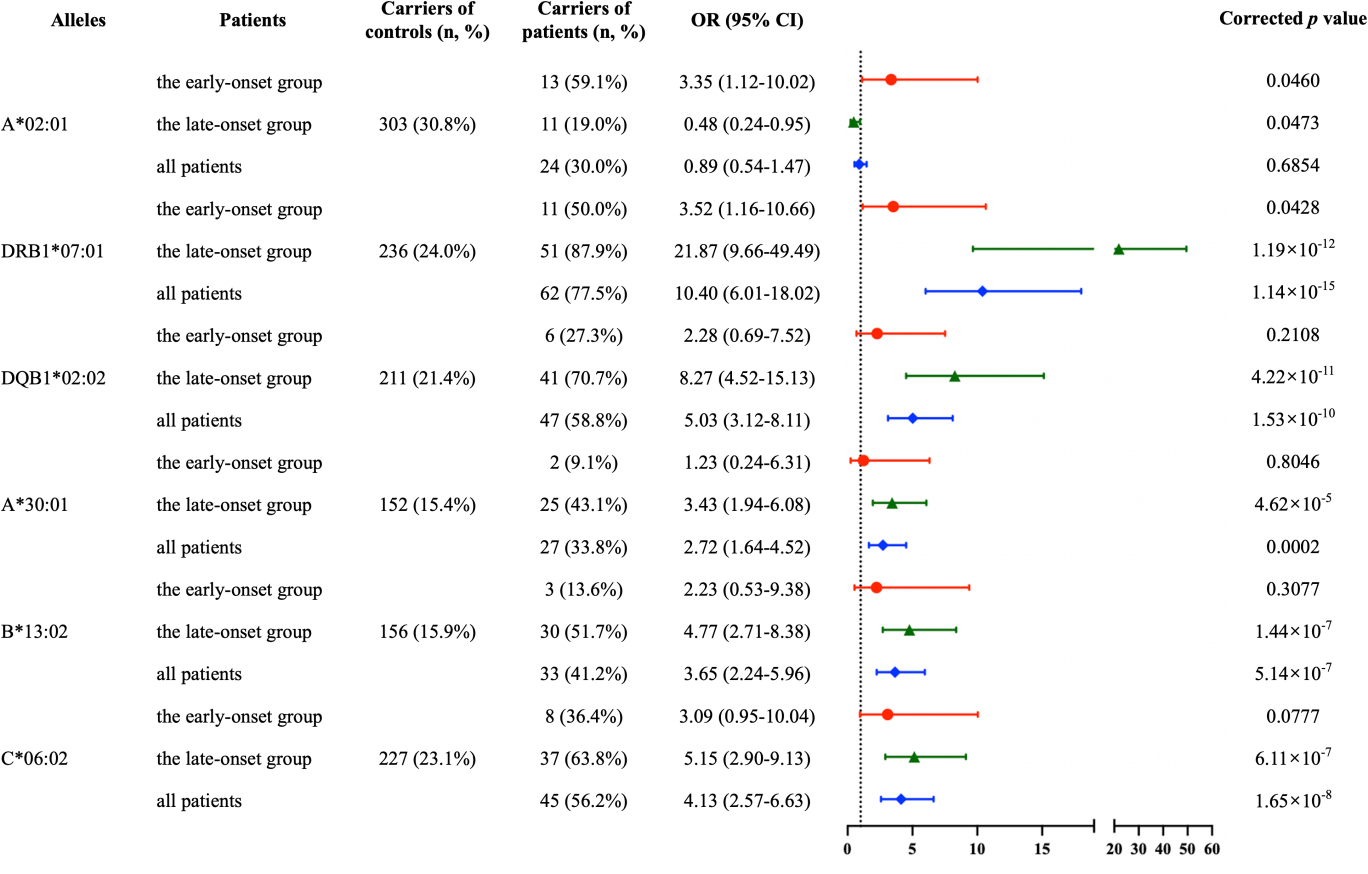
Association of HLA alleles with susceptibility to anti-LGI1 encephalitis in early-onset, late-onset, and overall patient populations. This forest plot illustrates the results of a logistic regression analysis examining the association between various HLA alleles and the susceptibility to anti-LGI1 encephalitis in a Chinese cohort. The odds ratio (OR) and 95% confidence intervals (CI) are shown for each allele. Data are presented for three groups: the early-onset patient group (red circles), the late-onset patient group (green triangles), and the combined “all patients” group (blue diamonds). The table on the left provides the carrier frequencies for both controls and patients. The FDR-adjusted *p*-values are shown on the right, which were used to correct for multiple comparisons.

Given the strong influence of age of onset observed in the previous analyses, we next stratified patients into early-onset and late-onset groups and focused on the six alleles associated with age of onset (A*02:01, DRB1*07:01, DQB1*02:02, A*30:01, C*06:02, and B*13:02). In this stratified analysis, DRB1*07:01 was identified as the primary risk allele, conferring susceptibility in both the early-onset group (OR = 3.52, 95% CI 1.16-10.66, *p*^c^ = 0.043) and the late-onset group (OR = 21.87, 95% CI 9.66-49.49, *p*^c^ = 1.19×10^-12^). Notably, A*02:01 emerged as a significant risk allele in the early-onset group, consistent with its higher frequency in this subgroup and reinforcing its role as a genetic determinant of early disease onset (OR = 3.35, 95% CI 1.12-10.02, *p*^c^ = 0.046) (**Figure 1**).

### Treatment and prognosis

All patients (80/80, 100%) received acute immunotherapy. Of these, 52 (65%) were administered a combination of corticosteroids and IVIG, 19 (23.8%) received corticosteroids alone, and 9 (11.3%) were treated with IVIG alone. Chronic immunotherapy was used in 75 patients, all of whom received mycophenolate mofetil (MMF) as initial (21). Among these, 20 patients subsequently transitioned to rituximab as escalated treatment, primarily due to inadequate response to initial immunotherapy. This treatment escalation was observed in 8 patients (36.4%) in the early-onset group and 12 patients (20.7%) in the late-onset group (*p* = 0.161).

The median follow-up duration for the entire cohort was 29 months (IQR 18-43.5 months). At the 12-month follow-up, 68 (85%) patients experienced improvement,10 (12.5%) were stable, and 2 (2.5%) patients deteriorated. The majority of patients (71.3%, 57/80) had attained favorable neurologic function (defined as mRS score 0-2), with scores of 0, 1, and 2 observed in 2, 31, and 24 patients, respectively. In contrast, 23 patients (28.8%) had an unfavorable outcome (mRS score >2), indicative of a poor prognosis (**Supplementary Figure 2**). Univariate analysis identified 8 factors associated with poor prognosis: diagnostic delay (OR = 1.01, 95% CI 1.00-1.01, *p* = 0.029), psychiatric symptoms (OR = 5.13, 95% CI 1.77-14.88*, p* = 0.003), FBDS (OR = 2.98, 95% CI 1.09-8.19, *p* = 0.034), GTCS (OR = 3.14, 95% CI 1.12-8.82, *p* = 0.030), sleep disorders (OR = 3.83, 95% CI 1.34-10.95, *p* = 0.012), autonomic symptoms (OR = 3.43, 95% CI 1.14-10.29, *p* = 0.028), hyponatremia (OR = 6.11, 95% CI 2.10-17.82, *p* = 0.001), mRS score at onset (OR = 5.96, 95% CI 2.25-15.78, *p* < 0.001) (**Supplementary Table 4**). No significant differences were observed in HLA allele frequencies between patients with good and poor prognoses (data not shown). Multivariate analysis was performed based on the above variables with significant differences in univariate analysis. The results identified diagnostic delay (OR = 1.01, 95% CI 1.00-1.02, *p* = 0.037) and GTCS (OR = 5.01, 95% CI 1.11-22.66, *p* = 0.036) as factors associated with poor prognosis (**Table 4**). Given the significant difference in mRS score at onset between the two groups, we further analyzed the change in mRS scores (Δ mRS) from baseline to the 12-month follow-up. The median Δ mRS was 1.0 (IQR 1.0-2.0) in the early-onset group and 1.0 (IQR 1.0-2.0) in the late-onset group, with no significant difference observed between the two groups (*p* = 0.669).

**Table 4:** Multivariate analysis of factors associated with poor prognosis.

Variables	OR (95%CI)	*P* value
Diagnostic delay (increase of 1 d)	1.01 (1.00 - 1.02)	0.037
Psychiatric symptoms	2.05 (0.42 - 10.09)	0.379
GTCS	5.01 (1.11 - 22.66)	0.036
FBDS	2.53 (0.63 - 10.17)	0.191
Sleep disorders	0.77 (0.13 - 4.60)	0.774
Autonomic symptoms	1.31 (0.21 - 8.08)	0.770
Hyponatremia	1.84 (0.40 - 8.36)	0.432
mRS score at onset	3.31 (0.89 - 12.27)	0.073

FBDS, faciobrachial dystonic seizures; GTCS, generalized tonic-clonic seizures; mRS, modified rankin scale.

## Discussion

In this single-center cohort study, we conducted a comprehensive analysis of HLA associations, clinical characteristics, and prognostic factors in Chinese patients with anti-LGI1 encephalitis. Our key finding is that specific HLA alleles not only confer susceptibility but also distinctly modulate the age of onset, thereby shaping clinical presentations. We confirmed DRB1*07:01 as the primary risk allele and identified DRB1*09:01 and DQB1*03:03 as novel, independent secondary risk factors in this population. Crucially, A*02:01 correlated with an earlier onset and was characterized by GTCS as the initial manifestation, whereas DRB1*07:01 was linked to later onset.

Our data affirm the prominent role of DRB1*07:01 as a major susceptibility allele in anti-LGI1 encephalitis. This finding aligns with reports from Korean and Caucasian populations (5, 11, 12), suggesting that DRB1*07:01 may represent a shared primary susceptibility allele across populations. Notably, the identification of the highly associated haplotype (A*30:01-C*06:02-B*13:02-DRB1*07:01-DQB1*02:02) in our cohort provides additional evidence supporting this conserved genetic association. However, this finding differs from previous reports in Southwest China (17), which identified DRB1*03:01 as the primary risk allele. This discrepancy likely reflects intra-ethnic genetic heterogeneity within the Han Chinese population, a hypothesis that warrants validation in larger, multi-center cohorts across China. Furthermore, our study underscores the significant heterogeneity in the genetic architecture of anti-LGI1 encephalitis across diverse ethnic groups, particularly concerning secondary risk loci. We identified DRB1*09:01 and DQB1*03:03 as significant risk alleles. Notably, in our Chinese cohort, these two alleles exhibit strong linkage disequilibrium, forming a DRB1*09:01-DQB1*03:03 haplotype. While DQB1*03:03 has been observed in Western cohorts, its contribution appears less defined; for instance, Mueller et al. reported an association in a Caucasian population (22), whereas Segal et al. found that the DQB1*03:03-containing haplotype did not reach statistical significance in an Israeli population (14). Crucially, unlike in Western populations where DQB1*03:03 is typically linked with DRB1*07:01, our data suggest that in the Chinese population it occurs within a haplotypic context driven by DRB1*09:01. In addition, DRB1*04:02, a well-established risk factor in Caucasian and Israeli cohorts (15), was not significantly associated with disease in our study. These population-specific associations may partly reflect differences in baseline allele frequencies across ethnic groups. In contrast to Western populations, the DRB1*09:01 allele is relatively common in East Asians, whereas DRB1*04:02 is comparatively rare, according to data from the Allele Frequency Net Database (AFND) (23). Mechanistically, it is conceivable that the HLA-DR molecules encoded by DRB1*09:01 and DRB1*07:01 may share certain structural or electrostatic features within their peptide-binding grooves, which may influence the repertoire of peptides presented to CD4^+^ T cells. Such differences in peptide presentation could affect immune recognition of LGI1-derived epitopes and thereby contribute to disease susceptibility. Further structural modeling and functional studies are required to elucidate the immunological basis of these population-specific associations.

Although anti-LGI1 encephalitis predominantly affects older individuals, early-onset patients were not rare (24). Our cohort exhibited a wide age at onset (15 to 83 years), with early-onset patients (younger than 50 years) constituting 27.5% of the total. The early-onset patients demonstrated a higher propensity for GTCS, while late-onset patients were predominantly characterized by amnesia and psychiatric symptoms. Crucially, the early-onset and late-onset groups displayed distinct profiles of HLA genes carriage. Specifically, we found that the presence of the A*02:01 allele was associated with a significantly earlier median onset age. Conversely, the strongest susceptibility allele, DRB1*07:01, was correlated with a markedly later onset. Our study reveals that HLA genes not only influence the risk of developing anti-LGI1 encephalitis but also critically modulate the age of disease onset, a phenomenon paralleled in other autoimmune diseases such as Type 1 Diabetes Mellitus (T1DM) and Myasthenia Gravis (MG) (25, 26). Nevertheless, the precise immunologic mechanisms underlying these observed differences remain to be elucidated.

Beyond susceptibility, prognostic factors are essential for clinical risk stratification. We found that both diagnostic delay and the presence of GTCS were independent predictors of poor functional prognosis in our cohort. While no specific HLA gene was significantly associated with poor prognosis in our analysis, this could be due to the limited sample size and the high polymorphism of HLA. Intriguingly, a recent retrospective study identified non-carrier status of DRB1*07:01 as an independent factor associated with unfavorable outcomes (24). This suggests that patients who do not carry DRB1*07:01(a profile more common in the early-onset group) might benefit from a more aggressive immunotherapy approach. Beyond genetic factors, there is growing interest in biomarkers for risk stratification. For instance, the acute-phase CSF/serum IL-6 ratio and serum IL-35 levels have been linked to disease severity and poor outcomes, respectively (27). Elevated serum neurofilament light chain (NfL) levels at onset show promise in identifying patients at higher risk of long-term cognitive sequelae (28). Collectively, these findings highlight the potential of integrating immunologic and neuronal biomarkers to guide clinical management, specifically in identifying patients (e.g., those who are DRB1*07:01 non-carriers or have elevated NfL) who might benefit from earlier, or more intensive, second-line immunotherapies.

This study is subject to several limitations. First, the limited sample size constrains the statistical power of the analysis, particularly within subgroups of carriers of rare alleles. Moreover, this limitation precluded the exploration of potential epistatic interactions between HLA alleles (e.g., using generalized multifactor dimensionality reduction [GMDR]), which could otherwise offer deeper insights into the complex genetic architecture of the disease. Second, while the mRS score is widely used for disability assessment in other studies of autoimmune encephalitis, its specificity for evaluating cognitive function and seizures is relatively limited, thus failing to comprehensively reflect the full scope of the patients’ condition. Furthermore, the relatively short follow-up period prevented a thorough evaluation of long-term recurrence rates and cognitive recovery. Finally, due to the rarity of anti-LGI1 encephalitis, replication in independent cohorts and functional validation using cell-based assays were not feasible in the present study. Future work is needed to confirm our findings and explore the underlying immunological mechanisms.

In conclusion, our study reveals the synergistic effects of HLA Class I and Class II genes in anti-LGI1 encephalitis, which not only influence disease susceptibility but also modulate the age of onset. DRB1*07:01 serves as the primary risk allele, whereas DRB1*09:01 and DQB1*03:03 constitute secondary risk alleles specific to the Chinese population. A*02:01 is associated with earlier onset, while DRB1*07:01 drives later onset and constitutes a major risk background. These findings provide insights into the role of HLA genotyping in accounting for the observed differences between early-onset and late-onset patients, and contribute to the realization of personalized diagnosis and treatment.

## Data Availability

The data presented in the study are deposited in the CNCB database (https://www.cncb.ac.cn/), accession number PRJCA060760.
