# Supplementary material for: HLA genotyping and clinical characteristics of early-onset and late-onset anti-LGI1 encephalitis: a single-center cohort study in China
Source: Front Immunol. 2026 Apr 1;17:1758914. doi: 10.3389/fimmu.2026.1758914 (PMC13079617; doi:10.3389/fimmu.2026.1758914)
Supplement: Supplementary file 1 [file Supplementaryfile1.docx]

**HLA genotyping and clinical characteristics of early-onset and late-onset anti-LGI1 encephalitis: a single-center cohort study in China**

Pinfei Ni^1^, Siyuan Fan^1^, Han Zhang^2^, Lin Bai^1^, Zhuo Yang^2^, Xinzhuang Yang^3^, Haitao Ren^1^, Qiang Lu^1^, Meng Xiao^2*^, Hongzhi Guan^1*^

**Author affiliations:**

^1^Department of Neurology, Peking Union Medical College Hospital, Chinese Academy of Medical Sciences & Peking Union Medical College, Beijing, China

^2^Department of Laboratory Medicine, Peking Union Medical College Hospital, Chinese Academy of Medical Sciences & Peking Union Medical College, Beijing, China

^3^Center for bioinformatics, National Infrastructures for Translational Medicine, Institute of Clinical Medicine, Peking Union Medical College Hospital, Chinese Academy of Medical Sciences & Peking Union Medical College, Beijing, China

*These authors contributed equally as co-corresponding authors.

*Correspondence to: **Hongzhi Guan**

Department of Neurology, Peking Union Medical College Hospital, Peking Union Medical College and Chinese Academy of Medical Sciences, Beijing, China

E-mail: [pumchghz@126.com](mailto:pumchghz@126.com)

*Correspondence to: **Meng Xiao**

Department of Laboratory Medicine, Peking Union Medical College Hospital, Peking Union Medical College and Chinese Academy of Medical Sciences, Beijing, China

E-mail: cjtcxiaomeng@aliyun.com

| **Supplementary Table S1. Summary of whole-exome sequencing data for the control cohort (n = 984).** | | |
| --- | --- | --- |
| **Item** | **Mean ± SD** | **Range** |
| Total reads (M) | 113.17 ± 25.71 | 70.02 - 159.82 |
| Total bases (Gb) | 16.98 ± 3.86 | 10.50 - 23.97 |
| Q30 (%) | 93.73 ± 1.00 | 92.00 - 95.49 |
| Q20 (%) | 97.75 ± 0.43 | 97.00 - 98.50 |
| Mapping rate (%) | 95.78 ± 1.69 | 90.01 - 98.99 |
| Mean sequencing depth | 152.17 ± 34.80 | 90.00 - 219.00 |
| Fraction of target covered ≥10× (%) | 98.02 ± 0.61 | 96.31 - 99.00 |
| Fraction of target covered ≥20× (%) | 95.75 ± 1.60 | 91.89 - 98.00 |
| Fraction of target covered ≥30× (%) | 92.76 ± 2.81 | 85.85 - 97.00 |

| **Supplementary Table S2. Hardy-Weinberg equilibrium (HWE) test results for HLA loci in the control group.** | | | | |
| --- | --- | --- | --- | --- |
| **Locus** | **χ2 (Chi-square)** | **df (Degrees of Freedom)** | ***P* value** | **Significance** |
| HLA-A | 82.98 | 70 | 0.138 | NS |
| HLA-B | 165.16 | 141 | 0.080 | NS |
| HLA-C | 99.40 | 91 | 0.257 | NS |
| HLA-DRB1 | 149.05 | 144 | 0.369 | NS |
| HLA-DQB1 | 47.24 | 75 | 0.995 | NS |

| **Supplementary** Table S3. Logistic regression analysis of HLA DRB1-DQB1 haplotype in patients with different onset ages and healthy control participants. | | | | | | | | |
| --- | --- | --- | --- | --- | --- | --- | --- | --- |
| DRB1-DQB1 haplotype | Controls (n =984) | Cases (n = 80) | | | Model 1 | | Model 2 | |
|  |  | Overall cohort (n = 80) | early-onset group (n = 22) | late-onset group (n = 58) | OR (95% CI) | Corrected p value | OR (95% CI) | Corrected p value |
| 07:01-02:02 | 211 (21.4%) | 47 (58.8%) | 6 (27.3%) | 41 (70.7%) | 5.03 (3.12 - 8.11) | 8.47E-10 |  |  |
| 07:01-03:03 | 29 (2.9%) | 16 (20.0%) | 6 (27.3%) | 10 (17.2%) | 8.04 (4.07 - 15.91) | 2.56E-08 | 13.04 (6.30 - 26.98) | 1.34E-11 |
| 09:01-03:03 | 227 (23.1%) | 34 (42.5%) | 8 (36.4%) | 26 (44.8%) | 2.4 (1.49 - 3.85) | 2.50E-03 | 3.31 (1.99 - 5.51) | 5.90E-06 |
| 15:01-06:02 | 256 (26.0%) | 9 (11.2%) | 3 (13.6%) | 6 (10.3%) | 0.36 (0.18 - 0.74) | 3.27E-02 | 0.46 (0.22 - 0.94) | 3.40E-02 |
| **Model 1:** Adjusted for age and sex. | | | | | | | | |
| **Model 2:** Further adjusted for primary significant haplotype (DRB1*07:01-DQB1*02:02) in addition to all variables in Model 1. | | | | | | | | |

| **Supplementary Table S4. Univariate analysis of prognostic factors.** | | | | |
| --- | --- | --- | --- | --- |
| **Characteristic** | **Good prognosis (n = 57)** | **Poor prognosis (n = 23)** | **OR (95%CI)** | ***p* value** |
| **Men** | 30(52.6) | 16(69.6) | 2.06(0.74-5.76) | 0.170 |
| **Age of onset** |  |  | 2.19(0.65-7.38) | 0.205 |
| < 50 | 18(31.6) | 4(17.4) |  |  |
| ≥ 50 | 39(68.4) | 19(82.6) |  |  |
| **Median diagnostic delay** | 59.00(26.00-91.00) | 70.00(57.00-168.00) | 1.01(1.00-1.01) | **0.029** |
| **First symptom** |  |  | 1.17(0.78-1.76) | 0.435 |
| Psychiatric symptoms | 2(3.5) | 0(0.0) |  |  |
| Amnesia | 10(17.5) | 6(26.1) |  |  |
| FBDS | 12(21.1) | 4(17.4) |  |  |
| GTCS | 14(24.6) | 2(8.7) |  |  |
| Other seizures (CPS+SPS） | 19(33.3) | 11(47.8) |  |  |
| **Disease course** |  |  |  |  |
| Psychiatric symptoms | 10(17.5) | 12(52.2) | 5.13(1.77-14.88) | **0.003** |
| Amnesia | 44(77.2) | 22(95.7) | 6.50(0.80-52.94) | 0.080 |
| FBDS | 22(38.6) | 15(65.2) | 2.98(1.09-8.19) | **0.034** |
| GTCS | 24(42.1) | 16(69.6) | 3.14(1.12-8.82) | **0.030** |
| Other seizures (CPS+SPS） | 41(71.9) | 15(65.2) | 0.73(0.26-2.06) | 0.554 |
| Sleep disorders | 11(19.3) | 11(47.8) | 3.83(1.34-10.95) | **0.012** |
| Autonomic symptoms | 9(15.8) | 9(39.1) | 3.43(1.14-10.29) | **0.028** |
| Personality changes | 8(14.0) | 6(26.1) | 2.16(0.66-7.13) | 0.206 |
| **Hyponatremia** | 10(17.5) | 13(56.5) | 6.11(2.10-17.82) | **0.001** |
| **mRS score at onset** |  |  | 5.96(2.25-15.78) | **< 0.001** |
| mRS=1 | 2(3.5) | 0(0) |  |  |
| mRS=2 | 11(19.3) | 1(4.3) |  |  |
| mRS=3 | 37(64.9) | 9(39.1) |  |  |
| mRS=4 | 7 (12.3) | 12(52.2) |  |  |
| mRS=5 | 0(0) | 1(4.3) |  |  |
| **CSF anti-LGI1 antibody positive** | 29(76.3) | 17(85.0) | 1.76(0.42-7.40) | 0.441 |
| **Abnormal MRI** | 33(76.7) | 21(95.5) | 6.36(0.76-53.40) | 0.088 |
| **Acute immunotherapy** |  |  | 1.09(0.53-2.22) | 0.819 |
| IVIG alone | 7(12.3) | 2(8.7) |  |  |
| CS alone | 13(22.8) | 6(26.1) |  |  |
| IVIG+CS | 37(64.9) | 15(65.2) |  |  |
| **Abbreviations:** FBDS, faciobrachial dystonic seizures; GTCS, generalized tonic-clonic seizures; CPS, complex partial seizures; SPS, simple partial seizures; mRS, modified rankin scale; CSF, cerebrospinal fluid; MRI, magnetic resonance imaging; IVIG, intravenous immunoglobulin; CS, corticosteroids. | | | | |


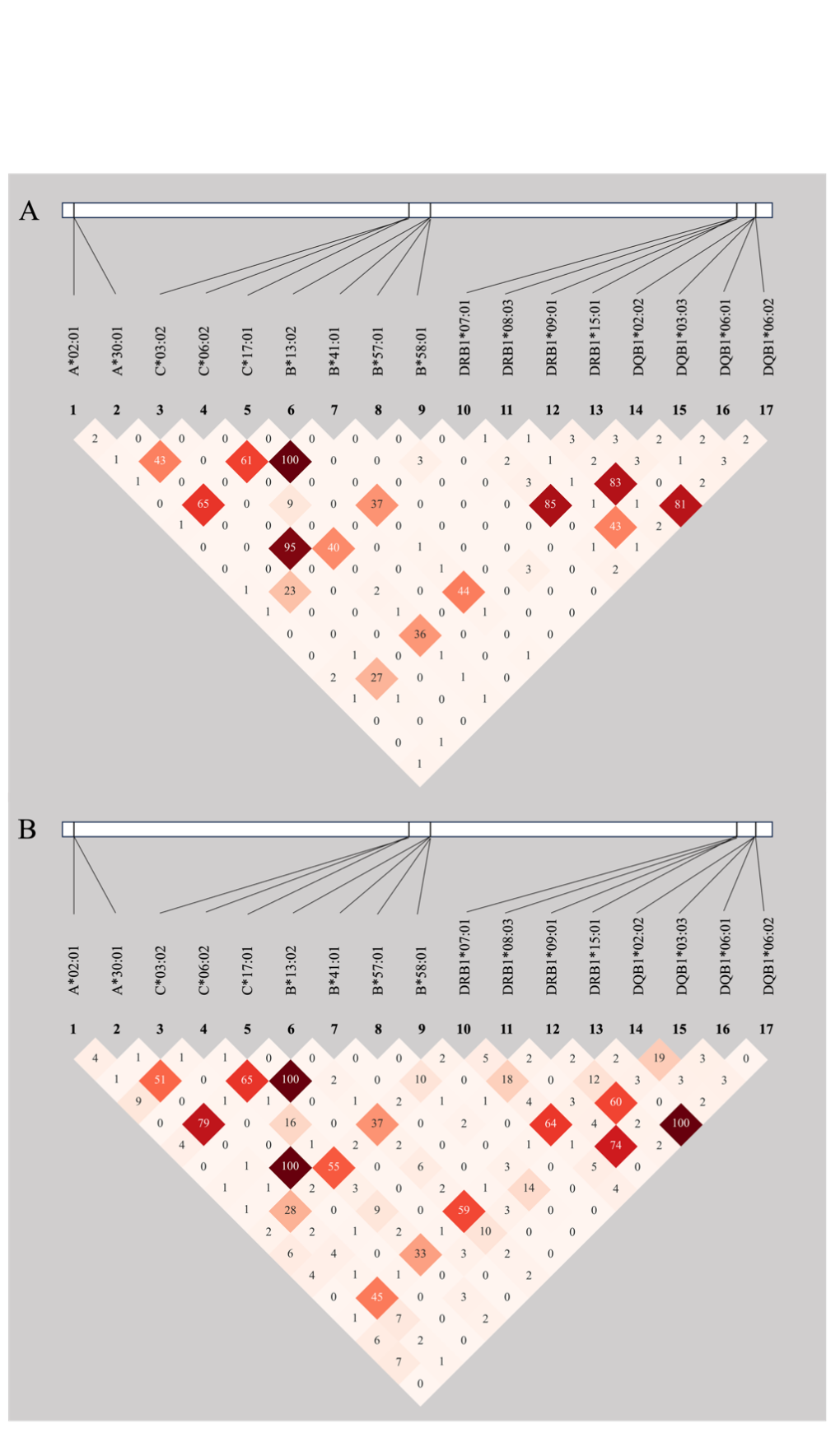


**Supplementary Fig. 1** Linkage disequilibrium (LD) plots for HLA alleles in healthy controls and patients with anti-LGI1 encephalitis.

Panel (A) presents the LD diagram for 984 healthy control participants, and Panel (B) shows the LD diagram for 80 patients with anti-LGI1 encephalitis. The numbers within each diamond represent the r^2^ ×100 value, a measure of LD between the two corresponding alleles. The intensity of the red color indicates the strength of the LD, with a darker red signifying a stronger linkage.


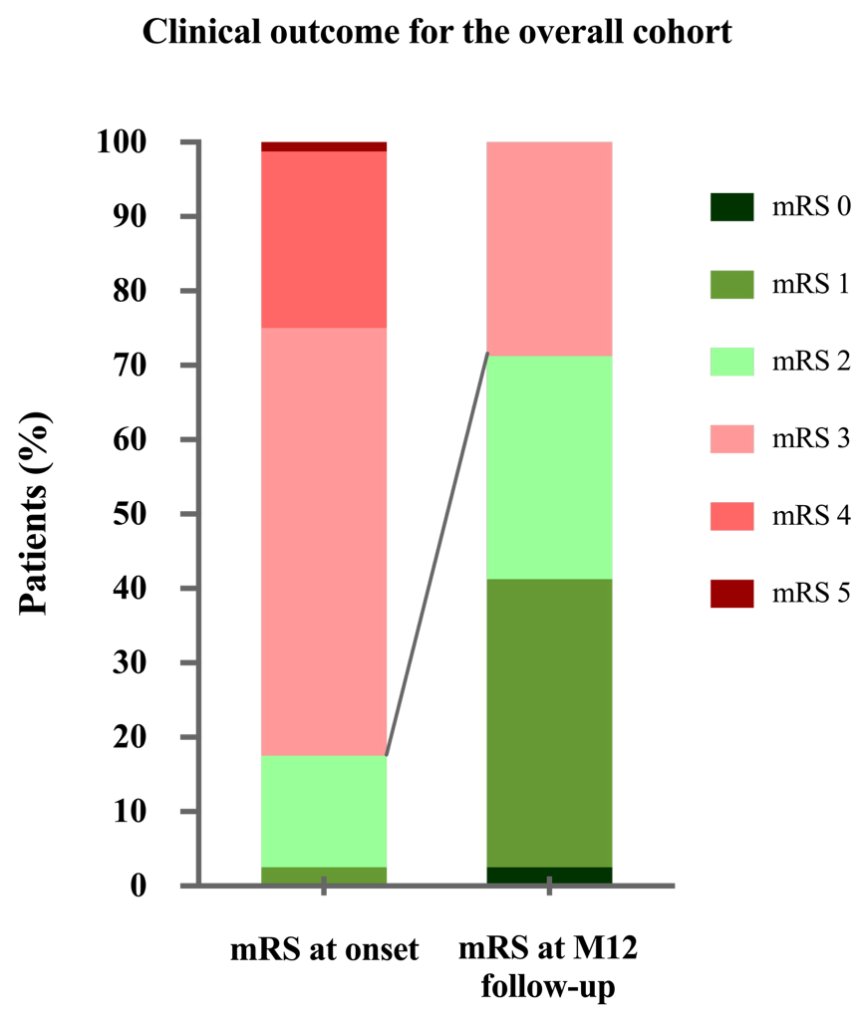


**Supplementary Fig. 2** Distribution of mRS scores at onset and the first 12-month follow-up.

mRS = modified Rankin Scale；M = month.
